# Selective antinociceptive effects of a combination of the *N*-methyl-D-aspartate receptor peptide antagonist [Ser^1^]histogranin and morphine in rat models of pain

**DOI:** 10.1002/prp2.32

**Published:** 2014-03-13

**Authors:** Aldric Hama, Jacqueline Sagen

**Affiliations:** Miami Project Cure Paralysis, University of Miami Miller School of MedicineMiami, Florida

**Keywords:** Combination therapy, endogenous antinociceptive neuropeptides, intrathecal drug delivery, synergism

## Abstract

Numerous rather than a few analgesic endogenous neuropeptides are likely to work in concert in vivo in ameliorating pain. Identification of effective neuropeptide combinations would also facilitate the development of gene or cell-based analgesics. In this study, opioid peptides endomorphin-1 (EM-1) and endomorphin-2 (EM-2) and the peptide histogranin analogue [Ser^1^]histogranin (SHG), which possess activity as an *N*-methyl-d-aspartate (NMDA) receptor antagonist, were intrathecally (i.t.) injected alone and in combination in rat models of acute and persistent pain. None of the peptides when injected alone altered hind paw responses of uninjured rats to acute noxious stimulation. EM-1 and EM-2 showed divergent efficacies in the persistent pain models. For example, EM-1 injected alone was antinociceptive in rats with neuropathic pain, whereas EM-2 demonstrated no efficacy. Demonstration of synergism was also divergent across the models. For example, while SHG combined with EM-1 did not alter the efficacy of EM-1 in rats with neuropathic pain, SHG significantly increased the efficacy of EM-1 in the formalin test. By contrast, the potency and efficacy of the peptides alone and combinations were much less than those of the reference analgesic morphine. Furthermore, morphine combined with the clinically used NMDA receptor antagonist ketamine showed synergism across a broad range of pain states. While the current set of neuropeptides could serve as a basis for analgesic therapeutics, there could be other neuropeptides with greater efficacy and potency and broader therapeutic application.

## Introduction

Chronic pain following nerve injury appears to be particularly resistant to commonly used acute pain therapeutics. Peripheral nerve injury leads to enhanced and persistent excitatory synaptic transmission within the spinal dorsal horn, in which the involvement of *N*-methyl-d-aspartate (NMDA) glutamate receptors found on spinal nociceptive neurons is particularly crucial (Coderre et al. 1993). These neurons in turn send nociceptive information to supraspinal neurons, some of which project back to dorsal horn neurons and further enhance activity of spinal cord neurons (Millan 1999). The spontaneous hyperactivity as well as hyper-responsiveness to peripheral stimulation of dorsal horn neurons in part underlies the spontaneous pain and cutaneous hypersensitivity to noxious and innocuous stimuli that occurs following nerve injury. In nerve-injured rats, the intrathecal (i.t.) administration of NMDA receptor antagonists decreases both abnormal activity of dorsal horn neurons and cutaneous hypersensitivity (Sotgiu and Biella 2000; Suzuki et al. 2001). These data suggest that patients with long-standing neuropathic pain would also benefit from i.t. injected NMDA receptor antagonists such as ketamine. However, the long-term effect of ketamine on human central nervous system tissue, the spinal cord in particular, has not been adequately addressed (Karpinski et al. 1997; Vranken et al. 2005). In addition, despite their presumed focused delivery into patients via the i.t. route, pyschomimetic side effects are obtained with analgesic doses of ketamine (Hawksworth and Serpell 1998).

Opioids have been shown to be efficacious in various long-term pain states, but one of the complications that arises is the need to gradually increase dosage due to a temporal increase in pain (Sallerin-Caute et al. 1998; van Dongen et al. 1999; Bennett et al. 2000). An alternate strategy to using either opioids or NMDA receptor antagonists alone would be to combine them, such that the doses of either drug are greatly decreased, yet the efficacy and the potency of the mixture are greatly enhanced with the added benefit of reduced seriousness of adverse side effects (Sator-Katzenschlager et al. 2001). In the long run, however, drug supply will need to be monitored and the intrathecal catheters will need to be carefully maintained in these patients.

In contrast to the short-term solution of a mechano-electrical delivery system, adrenal medullary chromaffin cells implanted into the subarachnoid space continuously secrete pain-reducing catecholamines, opioid peptides, and growth factors (Czech and Sagen 1995). In addition, chromaffin cells release the 15-amino acid peptide histogranin (HG), a NMDA receptor antagonist, which has been shown to be antinociceptive in rat models of chronic pain but has no effect on acute nociception, similar to small-molecule NMDA receptor antagonists (Lemaire et al. 1995; Siegan et al. 1997). Since numerous substances are released from the cells simultaneously, it is likely that the substances are working in concert.

Implanted chromaffin cells have demonstrated efficacy in chronic pain patients in small-scale studies (Winnie et al. 1993; Lazorthes et al. 1995). A significant technical hurdle, however, is the scarcity of human chromaffin cells and its heterogeneity from source to source, making it difficult to provide for consistent quantity and quality for clinical use. A possible solution would be to genetically enhance cells for transplantation with antinociceptive peptides. One candidate peptide would be the μ-opioid receptor selective endomorphins (EMs). EMs are good candidates since significant antinociceptive effects have been reported in both acute and chronic rodent pain models (Horvath et al. 1999; Przewlocka et al. 1999). Although chromaffin cells contain HG, a more stable analogue, [Ser^1^]histogranin (SHG), could be a better peptide for long-term studies. With increased cerebrospinal fluid (CSF) levels of both these peptides, it is possible that a pharmacological interaction between SHG and EM will lead to enhanced antinociception.

The main goal of this study is to characterize the behavioral effects of a combination of EM and SHG, in rat models of pain. The peptides were tested in several models because it is likely that sensitivity of the pain-related mechanism to the peptides will vary between models. For example, of i.t. opioid-mediated antinociception is enhanced in the inflamed compared to uninjured state, whereas neuropathic pain is thought to be insensitive to i.t. opioids (Hylden et al. 1991; Nichols et al. 1995). Furthermore, it is necessary to include a battery of endpoints. For example, while i.t. morphine may not have a significant effect on neuropathic tactile allodynia, it can attenuate neuropathic heat and cold hyperalgesia (Levy et al. 1994; Przewlocka et al. 1999). NMDA receptor antagonists also show varying effects depending on the model and behavioral endpoint (Yamamoto and Yaksh 1992b; Chaplan et al. 1997). For example, SHG attenuated inflammation-induced mechanical hyperalgesia better than thermal hyperalgesia (Hama and Sagen 2002). The effects of the peptides were compared with the effects of morphine and ketamine alone and in combination. This study demonstrate marked differences in effects, particularly in potencies, among the peptides alone and in combination among the various pain models.

## Materials and Methods

### Animals

Male Sprague–Dawley rats (225–275 g at the time of experiments; Harlan, Indianapolis, IN) were allowed 3–5 days to acclimate to the animal facility which was on a 12 h light/dark cycle. Prior to surgery and following surgery, rats were allowed free access to food and water. In an effort to reduce the number of rats undergoing surgery, rats were used twice (except in the formalin test) with a washout period of 2–3 days in between testing.

For surgical anesthesia, rats were rendered unconscious with 4–5% isofluorane in 100% O_2_ and maintained on 2–3% anesthesia. Aseptic technique was used throughout each surgical procedure. All procedures were reviewed and approved by the University of Miami Animal Care and Use Committee and followed the guidelines published by National Research Council (*Guide for the Care and Use of Laboratory Animals*).

### Intrathecal catheter surgery

Under isofluorane/O_2_ anesthesia, the atlanto-occipital membrane was exposed and a small cut was made (Yaksh and Rudy 1976). A sterile 8-cm catheter (32-g; ReCathCo, Allison Park, PA) was threaded down the intrathecal space toward the lumbar enlargement. The opposite end of the catheter was externalized through the skin on the back of the head and flushed with 10 μL of saline. The external catheter was melted shut. The muscle was sutured closed and the skin incision was sealed with veterinarian-grade cyanoacrylate. Rats were individually housed following i.t. surgery and allowed 3–5 days to recover. Upon completion of the behavioral experiments, 5 μL of 5% lidocaine was i.t. injected. Bilateral flaccid paralysis of the hind paws indicated proper catheter tip placement at the level of the lumbar enlargement. Following confirmation of catheter placement, the rats were euthanized by CO_2_ overdose.

### Chronic constriction injury

Under isofluorane/O_2_ anesthesia, the left sciatic nerve was exposed at the level of mid-thigh. Four 4-0 chromic gut ligatures were loosely tied around the sciatic nerve with about 1-mm spacing between them (Bennett and Xie 1988). The muscle wound was sutured shut and the skin was closed with veterinarian-grade cyanoacrylate. One week following chronic constriction injury (CCI) surgery, rats were implanted with an i.t. catheter as described. These rats were used for studies between 2–3 weeks after CCI surgery.

### Behavioral testing

Following baseline determination of either withdrawal latency or threshold, rats were i.t. injected with either vehicle (saline) or drug. Rats were tested once every 30 min for up to 120 min post i.t. injection.

In testing for either “thermal sensitivity” or “tactile sensitivity,” separate groups of CCI rats were used – individual CCI rats were not tested for both behavioral endpoints.

#### Thermal sensitivity

Rats were placed in Plexiglas chambers that rested on an elevated transparent surface (Hargreaves et al. 1988). Following 20–30 min of acclimation, a mobile infrared emitter was aimed at the center of the plantar hind paw. Activation of the emitter started a timer. A hind paw withdrawal from the surface terminated infrared stimulation and stopped the timer. The length of time between the onset of the stimulus and hind paw withdrawal was the withdrawal latency, measured in seconds. Three latencies were measured on each paw and in uninjured rats, and the final latency was reported as the average of both sides (average of six latencies). In nerve-injured rats, the average latencies are reported for each side (ligated vs. uninjured). About 5 min passed between stimulation of the same paw. To reduce the chance of skin damage to repeated heating, a cutoff of 20 sec was used.

#### Tactile sensitivity

Rats were placed in Plexiglas chambers that rested on an elevated wire grid surface. The 50% withdrawal threshold (reported in grams) was determined based upon the pattern of responses to von Frey filaments using the up-down method (Chaplan et al. 1994). Briefly, an ascending series of filaments were pressed at the center of the plantar hind paw until the rat withdrew its hind paw from the filament, then a lower force filament would be used until the rat stopped responding. A sequence of six responses and nonresponses were recorded and the 50% withdrawal threshold was calculated (Chaplan et al. 1994). The highest possible threshold was 15 g and the lowest possible threshold was 0.25 g. Nerve-injured rats with a withdrawal threshold of 4 g or less were included for study.

#### Formalin test

Rats were placed in Plexiglas containers and allowed to acclimate for 10–15 min. Following i.t. injection of either drug or vehicle, rats were replaced in the containers. Ten minutes after i.t. injection, 50 μL of phosphate-buffered 5% formalin was injected subcutaneously into the plantar left hind paw. Immediately following formalin injection, the number of hind paw flinches and licks in 1 minute were counted. Subsequently, formalin-evoked behaviors within 1 minute were recorded every 5 min up to 61 min after formalin injection. Following testing, rats were euthanized.

### Drugs

Rats were i.t. injected with either vehicle (saline) or a dose of morphine, EM-1, EM-2, SHG or ketamine in a volume of 5 μL followed by a 5-μL vehicle flush. See Table 1 for doses. The combination of opioid and NMDA receptor antagonist was i.t injected in a volume of 5 μL followed by a 5-μL vehicle flush. In the combination experiments, the dose of NMDA receptor antagonist was fixed, at either 10 μg ketamine or 0.3 μg SHG. The ineffective doses of ketamine and SHG were chosen based on previous studies (Siegan and Sagen 1997; Siegan et al. 1997; Hama et al. 2006).

**Table 1:** Doses (μg) of intrathecally injected opiates and NMDA receptor antagonists.

	Test			
	Acute/heat	CCI/heat	CCI/tactile	Formalin
Opiates				
Morphine	1, 3, 10	0.3, 1, 3, 10	0.1, 0.3, 1, 3	0.3, 1, 3
EM-1	3, 10, 30	1, 3, 10, 30	3, 10, 30	3, 10, 30
EM-2	20	3, 10, 30	2.5, 3, 8, 10, 25 30	1, 2.5, 3, 8, 10, 25, 30
NMDA receptor antagonists				
Ketamine	10	10	10	10
SHG	1	0.3	0.3	0.3

CCI, chronic constriction injury; EM-1, endomorphin-1; EM-2, endomorphin-2; NMDA, *N*-methyl-d-aspartate; SHG, [Ser^1^]histogranin.

Morphine sulfate, EM-1 and EM-2 were obtained from Sigma-Aldrich, Co. (St. Louis, MO) and SHG was custom synthesized by Bachem (Torrance, CA). Ketamine HCl (Generamedix Co., Liberty Corner, NJ) was obtained through our pharmacy. All drugs and peptides were dissolved in normal saline.

### Statistics

Statistical analysis was performed using SigmaStat 3.11. To compare the effect of treatments over time, a repeated-measure two-way repeated analysis of variance (ANOVA) was done, followed by a Student-Newman-Keuls post hoc test. Statistical significance was taken at *P* < 0.05. Number of rats = 6–7 per treatment group.

Dose–effect curves were obtained by converting the withdrawal thresholds obtained from rats with a CCI to a percent maximum possible effect (MPE):

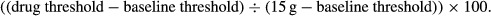


Similarly, the withdrawal latencies from both CCI and naïve rats were converted to a percent MPE:

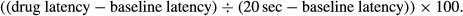


In the formalin test, there are two distinct phases of pain-related behaviors. Phase 1 was defined as the first minute phase following formalin injection and the phase 2 was defined as the 15–61 min phase following formalin injection. Total formalin-evoked behaviors in phase 1 and phase 2 were converted into a percentage of the maximum effect of vehicle:

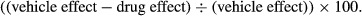


To determine if there was a change in antinociception with the combinations compared to the drugs alone, the potencies were calculated and compared. The 50% antinociceptive doses (A_50_) were calculated from the linear portion of the dose–response curves at 30 min post i.t. injection and the A_50_'s of the combinations were compared with the A_50_ of the drugs alone using a modified computer program (Tallarida and Murray 1981). Ninety-five percent confidence limits (95% CL) were also calculated. The typical isobolographic analysis was not used in this study since one of the drugs of the combination (NMDA receptor antagonists) was entirely ineffective and the dose was fixed (Porreca et al. 1990). Enhancement with the combinations was confirmed if a statistically significant leftward shift of the dose–response curve of the combination was observed compared to the dose–response curve of the effective drug (unpaired *t*-test, *P* < 0.05). The data are presented as mean ± SEM.

## Results

### Heat sensitivity

Prior to i.t. injection, baseline withdrawal latencies of the left and right hind paws of uninjured rats were similar (left = 11.2 ± 0.4 sec, right = 11.5 ± 0.5 sec). Since the side-to-side latencies were not different in naïve rats, drug effects of the left and right hind paws were combined and the average of the two is presented.

There was a significant interaction between i.t. drug treatment over time (*F*(20, 108) = 2.552, *P* = 0.0011). Post hoc analysis indicated a significant effect of morphine over time compared to vehicle treatment (below).

#### Acute/uninjured

**Figure 1:**
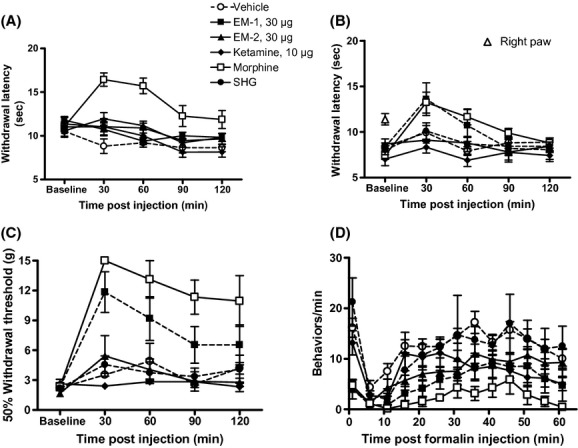
Effect of intrathecal injection of drugs in pain-related behaviors in rats over time. The highest doses of drugs injected alone used in the study are shown. (A) Acute/heat: Following baseline latency determination, rats were i.t. injected with either vehicle or drug in a volume of 5 μL. Rats were tested once every 30 min up to 120 min post injection. The latencies of the left and right hind paws were combined. (□) Dose of morphine = 10 μg. (●) Dose of SHG = 1 μg. (B) CCI/heat: Following baseline latency measurement in rats with a CCI, either drug or vehicle was i.t. injected and rats were tested once every 30 min up to 120 min post injection. The baseline withdrawal latency of the contralateral right hind paw (△) was not significantly different from the latency of the left hind paw prior to CCI. (□) Dose of morphine = 3 μg. (●) Dose of SHG = 0.3 μg. (C) CCI/tactile: Following baseline threshold determination, rats were i.t. injected with either drug or vehicle and tested once every 30 min up to 120 min post injection. (□) Dose of morphine = 3 μg. (●) Dose of SHG = 0.3 μg. (D) Formalin test: Either drug or vehicle was i.t. injected, followed 10 min later by 5% formalin injected into the left hind paw. The number of pain-related behaviors per minute was counted every 5 min until 61 min post formalin injection. (□) Dose of morphine = 3 μg. (●) Dose of SHG = 0.3 μg. Data are mean ± SEM. *n* = 6–7 rats per group.

**Figure 2:**
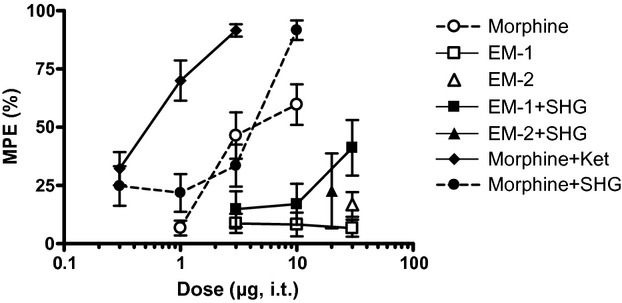
Effect of intrathecal injection of combination of drugs on acute noxious heat stimulation. The effects of the drugs at 30 min post injection are shown. The vertical axis is maximum possible effect (%) and the horizontal axis is dose (μg) of the opioid. For SHG, a fixed dose of 1 μg was combined with morphine and a dose of 0.3 μg was combined with EM. The dose of ketamine was fixed at 10 μg. Data are mean ± SEM. *n* = 6–7 rats per group.

Intrathecal injection of either EM-1 or EM-2 did not increase withdrawal latencies in uninjured rats (Figs. 1A, 2). By contrast, significantly elevated latencies were observed for 10 μg morphine at 30 and 60 min after i.t. injection (*P* < 0.05 vs. vehicle and baseline; Table 2, Fig. 2). There was no significant effect of either 1 μg SGH or 10 μg ketamine alone compared with vehicle-treated rats (Fig. 1A) at any time point following i.t. injection.

**Table 2:** Summary of 50% antinociceptive values and (95% confidence limits).

	Test				
	Acute/heat, μg (95% CL)	CCI/heat, μg (95% CL)	CCI/tactile, μg (95% CL)	Formalin, μg (95% CL)	
Drug				Phase 1	Phase 2
EM-1	0	>30 (16.4–130)	13.5 (6.2–29)	12.1 (3.8–38.7)	40 (14.6–107)
EM-1 + SHG	>30 (12.2–122.3)†	>30 (30–200)	13.9 (7.8–25)	1.5 (0.7–3)†	11 (5–22)†
EM-2	0	0	0	2.5 (1.2–5.1)	0
EM-2 + SHG	0	0	>30 (21–102)‡	4 (1.6–10.3)	0
Morphine	5.4 (3.6–7.9)	8 (3.4–18.5)	0.6 (0.3–1.1)	1 (0.4–2.3)	1.5 (0.8–2.8)
Morphine + SHG	2.6 (1.6–4.1)*	3.3 (1.4–7.8)	0.2 (0.1–0.4)*	0.1 (0.04–0.5)*	0.5 (0.2–1.1)*
Morphine + ketamine	0.5 (0.4–0.7)*	0.9 (0.6–1.3)*	0.3 (0.2–0.6)	0.1 (0.05–0.2)*	0.002 (0.0005–0.008)*
SHG	0	0	0	0	0
Ketamine	0	0	0	0	0

CL, confidence limits; CCI, chronic constriction injury; EM-1, endomorphin-1; EM-2, endomorphin-2; SHG, [Ser1]histogranin.

Fifty percent antinociceptive values, in μg, were calculated from the linear portion of the log dose–response curves (see Materials and Methods section). Values for acute/heat, CCI/heat, CCI/tactile were obtained at 30 min post injection. In the formalin test, Phase 1 data were obtained immediately following formalin injection (0–1 min) and Phase 2 data were obtained 15–61 min following formalin injection. The doses of SHG tested were 0.3 and 1 μg and for ketamine was 10 μg (see Table 1).

† *P* < 0.05 versus EM-1 alone;

‡ *P* < 0.05 versus EM-2 alone;

* *P* < 0.05 versus morphine alone.

Addition of either 10 μg ketamine or 1 μg SHG significantly increased the potency (A_50_) of morphine (*P* < 0.05 vs. morphine alone; Table 2). There was a 10-fold and twofold leftward shift in potency with the addition of either ketamine or SHG, respectively.

Furthermore, addition of either ketamine or SHG increased efficacy (% MPE). The efficacy of the combination of 3 μg morphine + 10 μg ketamine (91.7 ± 3.9% MPE) was significantly greater than that of 3 μg morphine alone (45.5 ± 15.2% MPE; unpaired *t*-test, *P* < 0.05). The efficacy of 10 μg morphine+1 μg SHG (90.8 ± 8.1% MPE) was also significantly increased compared to the efficacy of 10 μg morphine alone (59.7 ± 8.7% MPE; unpaired *t*-test, *P* < 0.05).

By comparison, the potency of the combination of 30 μg EM-1 + 1 μg SHG, compared to EM-1 alone, increased in uninjured rats (*P* < 0.05 vs. EM-1 alone; Table 2). Addition of SHG to EM-2 did not change either the potency or efficacy (% MPE) of EM-2.

#### CCI/heat

**Figure 3:**
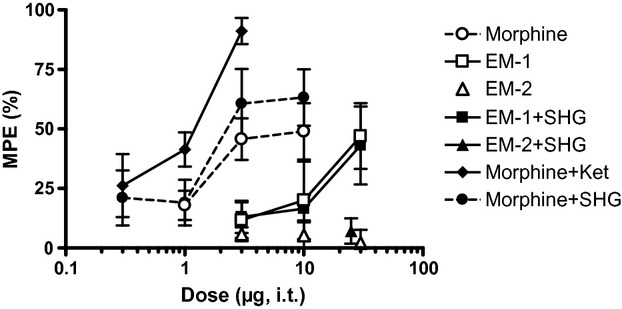
Effect of intrathecal injection of drug combinations on acute noxious heat stimulation in rats with a CCI. The percent maximum possible effects (MPE) of the drugs at 30 min post injection are shown. A fixed dose of either 0.3 μg SHG or 10 μg ketamine was used. The vertical axis is maximum possible effect (%) and the horizontal axis is dose (μg) of the opioid. Data are mean ± SEM. *n* = 6–7 rats per group.

Following nerve injury, withdrawal thresholds of the left ligated side (7.6 ± 0.3 sec) decreased compared to latencies of the right uninjured side (10.8 ± 0.3 sec; *P* < 0.05 vs. contralateral and presurgery latencies; Fig. 1B). There was a significant interaction between i.t. drug treatment and time (*F*(16, 116) = 3.249, *P* = 0.001). There was an increase in withdrawal latency with 30 μg EM-1 30 min after i.t. injection (*P* < 0.05 vs. baseline; Fig. 1B). Intrathecal injection of vehicle, 0.3 μg SHG, 10 μg ketamine, and EM-2 alone did not significantly alter latencies (*P* > 0.05; Figs. 1B, 3; Table 2). Addition of 0.3 μg SHG did not significantly alter the potency of either EM-1 or EM-2 (Table 2).

Morphine dose-dependently increased latencies of the ligated hind paw – a significant interaction was observed between i.t. morphine treatment and time (*F*(12, 96) = 2.170, *P* < 0.0191; Fig. 3). Interestingly, the efficacy of 10 μg morphine in the ligated hind paw (48.9 ± 11.8% MPE) was only slightly lower to that of uninjured rats (59.7 ± 8.7% MPE), despite the fact that the pre i.t. injection baseline latencies of CCI rats were much lower. The potencies (A_50_) in the injured (8 [3.4–18.5] μg) and uninjured rats (5.4 [3.6–7.9] μg) were not significantly different (*P* > 0.05; Table 2). Addition of SHG increased the potency of morphine twofold, but the shift was not statistically different from morphine alone in CCI rats (*P* > 0.05). Addition of 10 μg ketamine resulted in a significant ninefold leftward shift of the morphine dose–response curve (*P* < 0.05 vs. morphine alone in CCI rats; Fig. 3).

In addition to increased potency, the efficacy of the combination of morphine + ketamine in CCI rats was significantly increased compared to morphine alone. An i.t. dose of 3 μg morphine alone resulted in 45.7 ± 8.8% MPE. By contrast, the efficacy of i.t. 3 μg morphine + 10 μg ketamine was 91.1 ± 5.5% MPE and the combination of 3 μg morphine + 0.3 μg SHG was 60.7 ± 14.5% MPE. A one-way ANOVA comparing the efficacies of the morphine combinations to morphine treatment alone indicated a significant effect of ketamine (*P* < 0.05), but not SHG (*P* > 0.05), when added to 3 μg morphine (*F*(2, 17) = 4.46, *P* = 0.028; post hoc Dunnett's multiple comparison test).

### CCI/tactile sensitivity

Prior to nerve injury, withdrawal threshold of the left hind paw was 15 g. Following nerve injury, thresholds of the ligated hind paw decreased to 2.3 ± 0.2 g (Fig. 1C, “baseline”). Neither vehicle, 10 μg ketamine nor 0.3 μg SHG significantly altered withdrawal thresholds.

**Figure 4:**
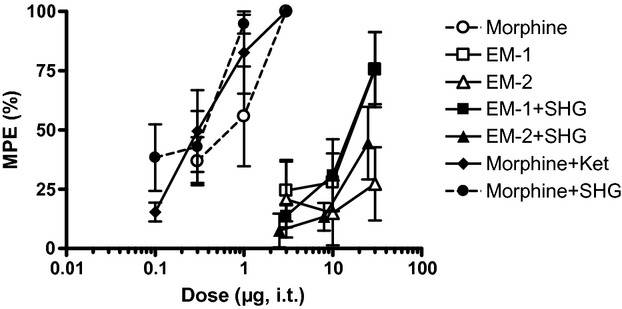
Effect of intrathecal injection of drug combinations to innocuous mechanical stimulation in rats with a CCI. The vertical axis is maximum possible effect (%) over time and the horizontal axis is dose (μg) of the opioid. Data are mean ± SEM. *n* = 6–7 rats per group.

There was a significant interaction between i.t. drug treatment and time (*F*(20, 124) = 3.929, *P* < 0.0001). Only the highest doses of EM-1 and EM-2 significantly increased thresholds (*P* < 0.05 vs. baseline; Fig. 4). Increased withdrawal thresholds began 30 min post i.t. injection for both EM-1 and EM-2. For EM-1, the antinociceptive effect lasted up to 120 min post injection. For EM-2, significant antinociception was not observed beyond 30 min post injection. In contrast, morphine dose-dependently reversed tactile hypersensitivity, beginning 30 min post injection and lasting for at least 120 min post injection (*F*(12, 84) = 2.522, *P* < 0.0069 vs. baseline; Fig. 1C).

The combination of 0.3 μg SHG with EM-1 did not enhance the antinociceptive effect of the opioid peptides (Table 2). However, the combination of 0.3 μg SHG with EM-2 was antinociceptive when EM-2 alone was not (*P* < 0.05 vs. EM-2 alone). The combination of morphine with either ketamine or SHG generally increased the potency of morphine. Specifically, the potency of the combination of SHG and morphine was increased by almost threefold, which was statistically significant compared to morphine alone (*P* < 0.05; Table 2). Although potency of the combination of ketamine and morphine was increased by about twofold, this was not statistically significant from that of morphine alone (*P* > 0.05).

### Formalin test

Figure 1D shows the effects of i.t. drug and vehicle treatment over time. A significant interaction was observed between i.t. drug treatment and time (*F*(60, 180) = 1.550, *P* = 0.008). For the purpose of constructing dose–response curves, total phase 1 and phase 2 behaviors were calculated. The mean total phase 1 and phase 2 pain-related behaviors of i.t. vehicle-treated rats were 15.2 ± 1.1 and 106.1 ± 5.9, respectively.

**Figure 5:**
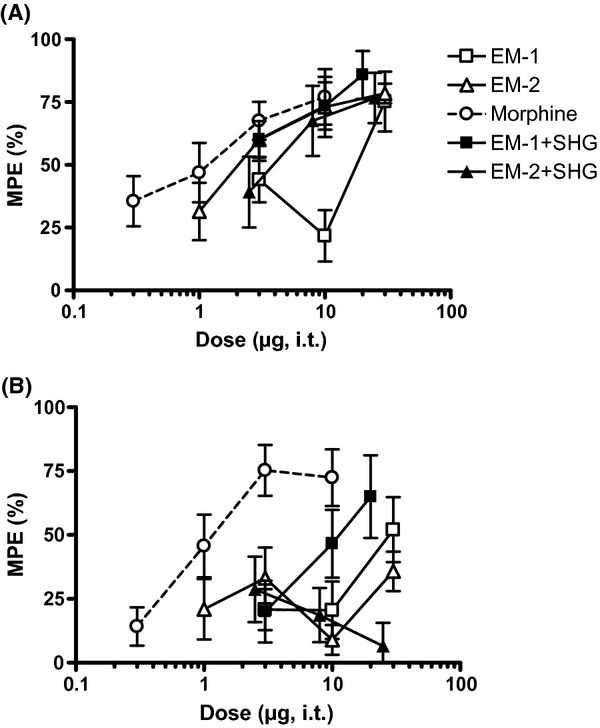
Effect of intrathecal injection of drug combinations in the formalin test. A fixed dose of 0.3 μg SHG and 10 μg ketamine was used. The vertical axis is maximum possible effect (%) and the horizontal axis is dose (μg) of the opioid. (A) Effect of the combinations on phase 1 (0–1 min post formalin injection). (B) Effect of the combinations on phase 2 (15–61 min post formalin injection). Data are mean ± SEM. *n* = 6–7 rats per group.

There was no significant effect following i.t. injection of either vehicle or 10 μg ketamine on formalin-evoked behaviors (Fig. 1D). Although not statistically significant, increased phase 1 pain-related behaviors were observed with i.t. 0.3 μg SHG (19.8 ± 3.5) compared to i.t. vehicle-treated rats (15.2 ± 1.1). Intrathecal injection of either morphine, EM-1 or EM-2 alone reduced phase 1 behaviors in a dose-dependent manner (*F*(5, 34) = 11.41, *P* < 0.001; Fig. 5A), but only morphine and EM-1 were able to reduce significantly phase 2 behaviors as well (*F*(5, 42) = 28.97, *P* *<* 0.0001; Fig. 5B).

Addition of 0.3 μg SHG significantly increased the potency of EM-1, eightfold in phase 1 and fourfold in phase 2, but not that of EM-2 (*P* < 0.05; Table 2). In fact, there was a trend toward diminished potency, possibly due to *increased* pain-related behaviors, in phase 1 in rats i.t. injected with EM-2 + SHG compared to rats i.t. injected with EM-2 alone, as the *right-ward* shift in the A_50_, from 2.5 to 4 μg, suggests.

By comparison, a previous study demonstrated a ninefold and over 100-fold increase in morphine potency in phase 1 and phase 2, respectively, with the addition of 10 μg ketamine (Hama et al. 2006). Also from that study, 0.3 μg SHG increased morphine potency sevenfold and threefold in phase 1 and phase 2, respectively (Table 2).

## Discussion

This study partially confirms previous findings of antinociceptive effects of i.t. injection of opioid receptor peptides EM-1 and EM-2 and compares their efficacy to the prototypical opioid receptor agonist morphine. The current data extend previous findings, showing that the addition of the peptide NMDA receptor antagonist SHG leads to improved opioid antinociception, but this improvement is dependent on the animal model, behavioral endpoint, and opioid. However, synergism with SHG cannot be generalized and was observed in specific pain models that is in contrast to the combination of morphine and ketamine in that enhancement was observed across almost all pain states. While the peptides evaluated in this study show some antinociceptive activity, perhaps other peptides and other combinations could show greater efficacy.

Adrenal chromaffin cells synthesize and secrete numerous antinociceptive substances including catecholamines, peptides, and trophic factors that could potently modulate nociceptive processing (Unsicker 1993). Previous studies have shown that the antinociceptive effects of subarachnoid chromaffin cell transplants are attenuated with the alpha-adrenoceptor antagonist phentolamine and opioid receptor antagonist naloxone (Hama and Sagen 1994). Previous studies have also shown that significant levels of norepinephrine and opioid peptide [Met]enkephalin are released from transplants into the rat spinal CSF long after transplantation surgery (Sagen and Kemmler 1989; Sagen et al. 1991). Thus, at least some of the antinociceptive effect is due activation of opioid receptors and adrenoceptors found within the spinal dorsal horn. Since these substances are continuously released into the spinal CSF, chromaffin cell transplants are an ideal therapeutic strategy for long-term pain treatment.

However, the scarcity and heterogeneity of human chromaffin cells could limit their wide-scale clinical application. In addition, it has not been established if natural chromaffin cells will be able to suppress a wide range of chronic pains. To address these issues, peptide genes could be expressed in chromaffin cells such that they release a greater variety of antinociceptive substances. In addition, greatly enhanced analgesia may be obtained by synergistic interactions, such as between opioids and NMDA receptor antagonists (Nishiyama 2000; Chow et al. 2004). Potential candidates for cell expression include EM and SHG.

Despite a superficial similarity of the peptides and the prototypic drugs in terms of their receptor targets, the effects of the drugs did not reliably predict efficacy of the peptides. While enhancement of morphine potency with ketamine was observed in all models except for tactile hypersensitivity in CCI rats, this study found an enhancement of EM potency with SHG under only limited conditions.

Intrathecal injection of EM was not antinociceptive to noxious heat in uninjured rats and others have noted a similar finding in the same test (Horvath et al. 1999). EMs appear to be antinociceptive in the tail–flick test although a wide range of efficacies have been reported (Horvath et al. 1999; Przewlocka et al. 1999; Hao et al. 2000). However, the onset and duration of EM antinociception does not appear to be entirely consistent – significant efficacy can either persist up to 60 min following injection or is entirely absent 30 min after injection (Horvath et al. 1999; Przewlocka et al. 1999; Hao et al. 2000). A significant effect of 10 μg EM-2 was reported to persist at least 120 min post injection (Przewlocka et al. 1999). However, about 50% of EM-2 is lost 40 min after incubation in rat brain homogenate, which suggests that efficacy lasting beyond this time point could be due to non-EM-2 mediated mechanisms (Janecka et al. 2006). Interestingly, Horvath et al. (2001) noted better efficacy of the EMs in the tail–flick versus hind paw heat test, though they did not speculate as to why this was so. The mechanism that underlies a differentiation of EM, between the tail–flick test and plantar hind paw heating, is unknown. The extent to which testing conditions influence efficacy of EM has yet to be elaborated.

The present data confirm an antinociceptive effect of i.t. EM-1 in rats with a CCI. A previous study, however, showed efficacy of EM-2 as well (Przewlocka et al. 1999). It is possible that the lack of EM-2 efficacy and diminished potency of EM-1 in this study, compared with Przewlocka et al. (1999), may be due to differences in injury etiology and sensory modality. Sensitivity to i.t. opioids in the neuropathic rats was confirmed in this study (and by Przewlocka et al.) with a dose-dependent morphine antinociception. Thus, the lack of EM-2 efficacy in this study cannot be attributed to a loss of dorsal horn opioid receptor responsiveness. In a different neuropathic model, the lack of i.t. morphine efficacy was attributed to its weak intrinsic efficacy (Nichols et al. 1995). That the combination of EM-2 + SHG led to antinociception in CCI rats could support this contention. However, it is possible that EM-2 works through other, nonopioid targets and that nerve injury led to the loss of those other targets.

In the formalin test, this study was in general agreement with previous results, in that EM-1 reduced both phase 1 and phase 2 pain-related behaviors (Przewlocka et al. 1999; Hao et al. 2000). Interestingly, in this study, EM-2 decreased phase 1 behaviors but not phase 2 behaviors. Depending on the drug, i.t. pretreatment either suppresses both phases or suppresses phase 2 greater than phase 1 (Yamamoto and Yaksh 1992a). There are no other examples in the literature that demonstrates a selective reduction in phase 1. The acute phase is generally believed to be the immediate nociceptive response to formalin, somewhat similar in mechanism to a brief thermal stimulus in the plantar heat test. It is possible that the formalin-induced acute pain and brief thermal stimulation could be mechanistically similar, but the current data does not support this contention. A study that compared the efficacies of i.t. injected compounds in the formalin test (acute phase) and the hot plate test showed no correlation (Malmberg and Yaksh 1994; Yamamoto et al. 1997). Thus, the two “acute” pains are mechanistically distinct rather than similar.

Although the literature suggests that i.t. co-administration of an NMDA receptor antagonist with a sub-effective dose of opioid leads to an increase in opioid efficacy, this is observed in limited experimental conditions (Hoffmann et al. 2003; Redwine and Trujillo 2003). Intrathecal NMDA receptor antagonists have no antinociceptive efficacy in uninjured rats (Nishiyama et al. 1998); a lack of effect of both 10 μg SGH and 30 μg ketamine (data not shown) was observed in the plantar thermal test. In this study, an enhanced antinociception was observed with a combination of EM-1 + SHG in the thermal test, which is consistent with a previous report showing enhanced EM-1 efficacy when combined with ketamine (Horvath et al. 2001). There is no report in the literature on the effect of a combination of EM-2 with an NMDA receptor antagonist. Since the NMDA receptor is inactive in the uninjured or unsensitized state, the enhanced effect of an NMDA receptor antagonist and opioid in an acute pain model must be through a mechanism that utilizes receptors other than the NMDA receptor (Headley et al. 1987).

In contrast to acute pain, NMDA receptor activity in the neuropathic state is crucial in diminishing opioid efficacy, since i.t. injection of NMDA receptor antagonists both suppress neuropathic pain and normalize the antinociceptive effect of i.t. morphine (Mao et al. 1995; Chaplan et al. 1997). A leftward shift in the opioid dose–effect curve indicates an additive/synergistic effect of the NMDA receptor antagonist. In this study, i.t. injection of a combination of morphine + ketamine in neuropathic rats led to a two- to ninefold increase in potency. Likewise, SHG led to a two- to threefold increase in morphine potency. As mentioned earlier, increased opioid potency with the addition of a NMDA receptor antagonist does not generalize to all ligands (Hoffmann et al. 2003). An enhancement of the efficacy of EM with SHG was observed only in specific pain states in contrast to morphine.

Although preclinical data generally support an enhancement of opioid antinociception with the addition of a NMDA receptor antagonist, it is still difficult to elaborate the exact mechanism for this effect since clinical trials as well as preclinical studies have not always demonstrated additivity/synergy (Choe et al. 1997; Wadhwa et al. 2001; Galer et al. 2005). Two generalizations can be made based on the current data. It appears that obtaining significant enhancement with an NMDA receptor antagonist depends on the ligand and the pain state. For example, ketamine binds to non-NMDA receptors and acetylcholine receptors as well as to NMDA receptors (Kress 1994; Gonzales et al. 1995). Although SHG may have interactions with other receptors, it appears that these interactions are insufficient to markedly increase morphine potency (Ruan and Lemaire 2001). Also, it appears that *preinjury* treatment with a combination leads to better efficacy, as well as potency, compared to either drug alone. Thus, therapeutic development should proceed in models that parallel the clinical condition in order to obtain an accurate picture of both potency and efficacy. Based on the current study, i.t. injection of a combination of NMDA receptor antagonist and opioid could yield an enhancement of morphine potency – and possibly efficacy – if given *before* an injury (Hama et al. 2006). The same i.t combination could also be useful in an ongoing pain state, but depending on the type of pain.

Future studies should evaluate the long-term i.t. infusion of the combinations, not only to mimic the clinical condition but also to partially mimic the action of transplanted cells or neurons transduced with novel peptide genes. In the case of chromaffin cells for pain relief, since they synthesize and secrete numerous substances, the potential exists for numerous novel combinations with significant clinical efficacy. Thus, it is possible that other combinations, such as SHG and trophic factors, could also be additive but show efficacy across a wider range of pain models.

## Abbreviations

CCI: chronic constriction injury
CL: confidence limits
CSF: cerebrospinal fluid
EM-1: endomorphin-1
EM-2: endomorphin-2
i.t.: intrathecal
MPE: maximum possible effect
NMDA: *N*-methyl-d-aspartate
SHG: [Ser^1^]histogranin
